# A year of One Health and neglected tropical diseases

**DOI:** 10.1093/inthealth/ihad043

**Published:** 2023-06-20

**Authors:** Gabrielle Laing

**Affiliations:** Unlimit Health, Edinburgh House, 170 Kennington Lane, London SE11 5DP, UK

***30 January 2023 saw World Neglected Tropical Disease (NTD) Day so we took a moment to reflect on the last year with the NTD NGO Network's (NNN) One Health Group*.**

World NTD Day January 2022^1^ saw the launch of what the WHO news called a ‘key document to guide a paradigm shift towards One Health’ in neglected tropical diseases (NTDs). This document was part high-level strategy and part user-guide to help inspire NTD programme teams where One Health could be utilised to their advantage. Fundamentally, One Health is about understanding ecosystem interactions and, where appropriate, bringing together relevant stakeholders and sectors to take a coordinated approach. For example, by designing interventions that address common human, animal or environmental risk factors, or that build core capacities such as surveillance to strengthen health systems overall.

One Health approaches are key to sustainably preventing NTDs because they recognise the interlinked nature of human, animal and environmental health, and take a ‘whole of system’ approach to action. The NTD Non-governmental organisation Network (NNN) One Health group co-authored the One Health for NTDs companion document (Figure 1), calling upon its network of non-state actors from around the world to help contribute their ideas and lessons learned. This allowed us to create a set of guiding principles for anyone, from any background, to consider when embracing a cross-cutting approach to NTDs. These principles encouraged those working in NTDs to explore where a more integrated approach would be suitable and beneficial and, as the foreword from Freetown's mayor, Yvonne Aki-Sawyerr OBE, says, to ‘Start now, start anywhere, with the context and resources available—then expand your approach as you build capability, connections, and momentum’.

**Figure 1.**
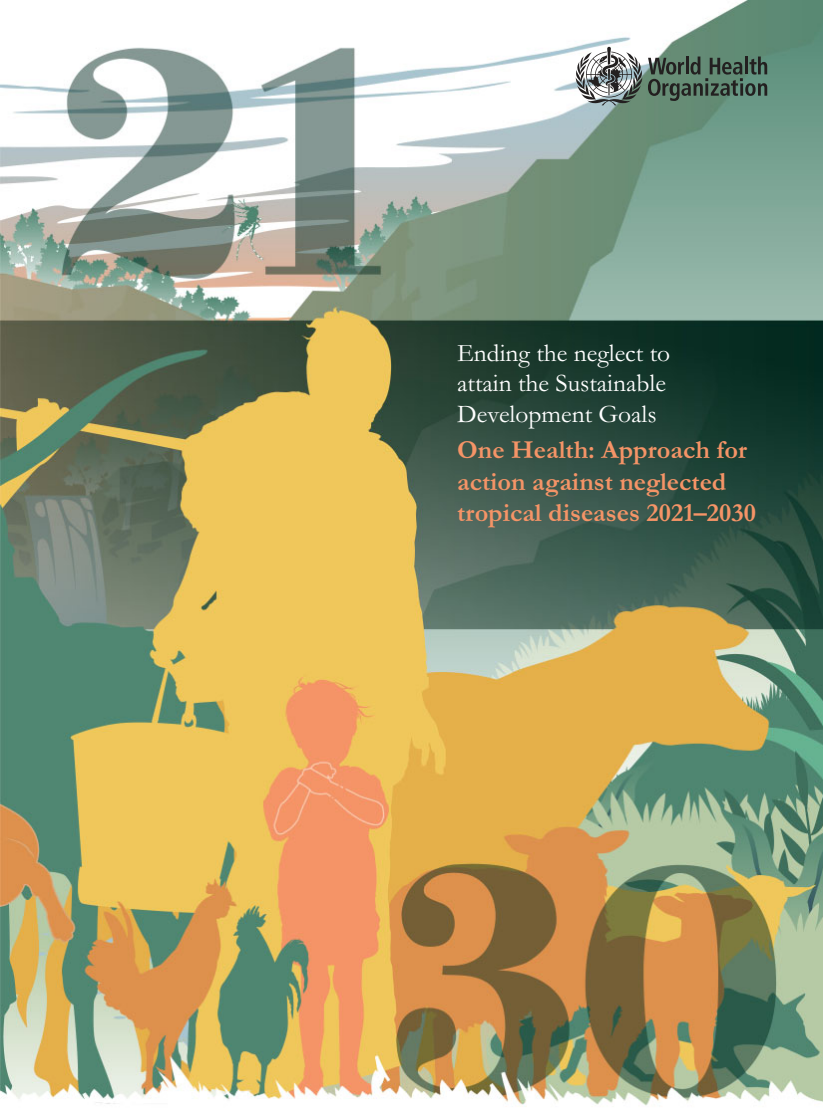
Front cover of the WHO's One Health approach for NTDs, a companion document to the 2021–2030 road map, co-authored by the NNN One Health group. (CC https://creativecommons.org/licenses/by-nc-sa/3.0/igo/deed.en Attribution-NonCommercial-ShareAlike 3.0 IGO (CC BY-NC-SA 3.0 IGO)).

The report launch was followed by a successful webinar^2^ with >900 live participants from 106 countries. Talks were on unlocking political will to drive progress and the World Bank speaking on sustainable One Health financing. Case studies of environmental interventions against NTDs and veterinary public health contributions in surveillance were also discussed, hosting speakers from Sierra Leone, Mauritius and Singapore. The chat box and Q&A sessions were vibrant with enthusiasm for the opportunities of shared advocacy between sectors, and a move towards preventative action.

Reflecting on the interest generated by the companion document and webinar launch, we at the NNN One Health group wanted to think about how to turn this into action. As a group, we are spread across Africa, Asia, Europe and the Americas, with those from animal health, vector control, water, sanitation and hygiene (WASH), academia and social sciences alongside human health expertise. This diversity of members can sometimes mean the language we use to communicate can differ, not only by country, but also by sector. However, we are all motivated by a common interest in One Health and a belief that it is critical in achieving the targets set by the NTD road map. So, at this year's NNN conference in Nepal we ran a workshop on just that.

The workshop invited speakers from the WHO zoonotic NTD team, regional South East (SE) Asia office and those with lessons learned from NTD interventions in Africa. Case studies highlighted the failure of Guinea Worm eradication programmes in Chad to account for domestic dogs as a reservoir of infection, and the sometimes conflicting wildlife–human interface. We also discussed environmental interventions enacted against NTDs across SE Asia and considered integrated taeniasis control strategies across food safety, WASH and agriculture.

This year, the NNN's wider focus is on enabling a shift to country-led action and the One Health group is looking forward to responding to countries’ interest in and demand for One Health. Our workshop's main aim was to ensure that One Health is seen as more than just collaborating with veterinary public health and that it is about examining the whole system surrounding NTDs, identifying where there needs to be integration, or where working across sectors can help to achieve goals more effectively or efficiently. Our next steps will help to identify what gaps and barriers we can help countries to overcome to fully operationalise One Health approaches against NTDs.

One Health as an approach has understandably received widespread interest in the wake of the COVID-19 pandemic. The Intergovernmental Negotiating Body of the WHO included a 3-h evidence session^3^ on One Health for the Pandemic Instrument, and the UK Government have promoted the approach in their Ending Preventable Deaths and Health Systems Strengthening papers.^4^ This year has also seen a flurry of activity from the Quadripartite alliance of agencies (Food and Agriculture Organization of the United Nations (FAO), the United Nations Environment Programme (UNEP), the World Health Organization (WHO), and the World Organisation for Animal Health (WOAH, founded as OIE)) and their One Health High Level Expert Panel, culminating with a Joint Plan of Action^5^ and a well-received Theory of Change.^6^ The Joint Plan of Action identifies key areas for NTDs alongside endemic and vector-borne diseases, with the continued development of data surveillance, management and information sharing, implementation of control activities, stakeholder training, (risk) communication and community engagement all promoted. The evidence base for taking a One Health approach to NTDs was similarly recognised by a recent Lancet series on One Health and Global Health Security.^7^

NTDs are a diverse group of diseases, similar only in their neglected status of those affected. They comprise non-communicable diseases, direct zoonoses, vector-borne diseases and those with environmental factors. It is this diversity that presents an opportunity for cross-cutting interventions to have a real impact.^8,9^

Sometimes, the One Health approach can seem very broad or a bit nebulous, making it a daunting path to embark upon. But, focusing on the NTDs as a starting point for a government, subnational body or civil society organisation might present a more accessible route in. Establishing One Health ways of thinking, working and financing will demand extra effort initially, but once institutionalised, these practices should be scalable beyond NTDs while still remaining adaptable enough for all local contexts, while keeping the community at the heart of everything.

## Data Availability

None.
